# Evaluation of medication regimen complexity and rehospitalization in older adults with chronic kidney disease: a retrospective cohort study

**DOI:** 10.1186/s12877-026-07986-7

**Published:** 2026-07-16

**Authors:** Aslınur Albayrak, Ceyda Nur Başarır, Atila Altuntaş

**Affiliations:** 1 Department of Clinical Pharmacy, Faculty of Pharmacy, Suleyman Demirel University, Isparta, Turkey; 2 Faculty of Pharmacy, Suleyman Demirel University, Isparta, Turkey; 3 Department of Nephrology, Faculty of Medicine, Suleyman Demirel University, Isparta, Turkey

**Keywords:** Anticholinergic cognitive burden scale, Chronic kidney disease, Medication regimen complexity, Older adults, Rehospitalization

## Abstract

**Background:**

Polypharmacy is quite common in older adults with chronic kidney disease (CKD), and these patients have complex medication regimens. The medication regimen complexity index (MRCI) is one of the tools most commonly used to assess medication regimen complexity. CKD patients experience recurrent rehospitalization. However, the relationship between the MRCI score and rehospitalization in CKD patients is unclear, and evidence on this subject is limited, particularly in older adults. Therefore, this study aims to evaluate the relationship between the MRCI score and rehospitalization within 30 days and 90 days in older adults with CKD patients.

**Methods:**

This retrospective study was conducted in older adults with CKD hospitalized in the nephrology service of Suleyman Demirel University Research and Practice Hospital, Isparta, Turkey, between August 2019 and August 2023. In this study, medication complexity was assessed using the MRCI score and anticholinergic burden was measured with the Anticholinergic Cognitive Burden Scale (ACBS). Multiple linear regression analysis was used to identify factors influencing the MRCI score. Binary logistic regression models were created to determine factors affecting 30-day and 90-day rehospitalization.

**Results:**

The mean (± SD) MRCI score of the patients was 23.4 ± 13.65. The factors affecting the MRCI score were the ACBS score, diabetes, thyroid diseases, hypertension; and rehospitalization within 90 days as a result of the forward selection method (*p* < 0.05).

According to logistic regression analysis, the MRCI score and diabetes significantly affected rehospitalization within 30 days and 90 days (*p* < 0.05).

**Conclusions:**

In this study, the ACBS score, diabetes, thyroid diseases, hypertension and rehospitalization within 90 days significantly affected the MRCI score. Diabetes and MRCI were factors affecting rehospitalization within 30 days and 90 days. Reducing medication complexity may be a factor that can prevent rehospitalization in older adults with CKD.

## Background

Chronic kidney disease (CKD) is a progressive disease and patients may suffer from comorbidities such as bone and mineral disorders, anaemia, electrolyte imbalance, and hypertension [1]. For this reason, CKD patients use multiple medications, called polypharmacy, to slow the course of their disease and treat additional complications [2].

Polypharmacy is quite common in older adults with CKD, with studies showing a prevalence between 62.0% and 86.0% [3, 4]. Research carried out in Turkey revealed a very high rate of polypharmacy. Pehlivanlı et al. reported a 92% rate of polypharmacy in their study of older adults with CKD patients, and Erken et al. reported a 74.4% rate [5, 6]. Studies have shown that polypharmacy in older adults with CKD patients are associated with an increased risk of CVD events and all-cause mortality [4, 7, 8]. As a result of polypharmacy these patients have complex medication regimens [3, 9]. Factors such as the number of medications used, dosage form, dosing frequency, and additional instructions for medication use contribute to the complexity of medication regimens [10, 11]. The medication regimen complexity index (MRCI) is one of the most commonly used tools to assess medication regimen complexity [11].

CKD patients have a high tendency to be hospitalized and have frequent rehospitalization [12–14]. Hospitalization is associated with increased morbidity and mortality and decreased quality of life [15, 16]. It also poses a significant emotional burden to patients and their caregivers [12]. Although hospitalization is common in patients with CKD, medication regimen complexity is a critical concern in older adults. Therefore, evaluating the association between medication regimen complexity and rehospitalization is particularly vital in such individuals [17]. There are studies that relate the complexity of medication regimens to clinical outcomes such as hospitalization and rehospitalization [10, 18]. While some studies have shown that the MRCI score is associated with rehospitalization [18, 19] other studies have not shown a relationship [20, 21]. In studies conducted in older adults with CKD patients, no relationship was found between the MRCI score and rehospitalization [9, 17]. Furthermore, studies on this subject are limited. This study aims to evaluate the relationship between the MRCI score and rehospitalization within 30 days and 90 days in older adults with CKD patients. Additionally, factors affecting the MRCI score were also examined.

## Methods

### Study design

This retrospective study was conducted in older adults with CKD patients hospitalized at the nephrology service of Suleyman Demirel University Research and Practice Hospital, Isparta, Turkey, between August 2019 and August 2023. There were three physicians in the service where the study was conducted. Patients with CKD (estimated glomerular filtration rate [eGFR] of 15–60 mL/min/1.73 m2) aged > 65 years were included in the study. Patients who were hospitalized for less than 24 h, patients who received renal replacement therapy, and patients whose data were insufficient were not included. The ethics committee decision was approved by the Suleyman Demirel University Faculty of Medicine Clinical Research Ethics Committee (Approval No:14/196 Date:31.10.2023).

Using the Raosoft sample size calculator, the sample size was calculated to be at least 128 with a 5% margin of error, a 95% confidence interval, and a 50% distribution ratio [22]. The sample size was calculated retrospectively to examine the power provided by the selected convenience sample. All patients who met the inclusion criteria were included in the study.

The demographic, laboratory and medical information of the patients was accessed through the hospital’s electronic medical record system. Data collection, MRCI calculation, and verification were performed by two independent researchers.

The outcome of this study was to determine the relationship between the MRCI score and 30-day and 90-day rehospitalization rates in older adults with CKD patients. All patients were based on their initial hospitalization during the study period, and the 30-day and 90-day rehospitalization rates were calculated as the time from the initial discharge date to the date of rehospitalization.

### Measures

#### Polypharmacy

The use of five or more medications was considered polypharmacy [23, 24].

#### Medication regimen complexity index (MRCI)

The MRCI was developed by George et al. [11]. The Turkish- validated version of the MRCI was used in this study. Permission to use the scale was obtained from the first authors of the study [11, 25].

This scale consists of 65 items that include information about dosage forms, dosing frequencies, and drug administration. Part A addresses dosage forms, part B addresses dosing frequency, and part C addresses additional instructions. The score increases as the drug dosage form becomes more complex, the daily dosing frequency increases, and additional instructions are added. The total score is determined by adding the scores of sections A, B and C. The MRCI score range and cut-off point are not definitive. While the minimum score is 1, there is no specific maximum score [11].

In this study, combination medications were considered a single medication due to their single dosage form and dosing frequency. However, any complexities associated with the combination (e.g., special instructions) were reflected as points in the corresponding MRCI section.

#### Anticholinergic drug use

Some studies have shown adverse effects of anticholinergic medication use in older adult patients, such as increased rehospitalization [26, 27]. Older adults with CKD patients frequently use anticholinergic drugs [28, 29]. In this study The Anticholinergic Cognitive Burden Scale (ACBS) was used to assess anticholinergic drug use [30]. Although many scales have been developed to determine anticholinergic burden, the ACBS is one of the scales with proven quality according to parameters such as precision of development, clarity of presentation and applicability [31]. Scale scores vary between 0 and 3 points. Scores are assigned based on whether the drug has a no, possible, or definite anticholinergic activity. The updated version of the ACBS was used in our study [32]. Permission to use the scale was obtained from the first author of the study [30]. An ACBS score above 1 was considered to be indicative of the presence of anticholinergic drug use.

### Statistical analysis

The Statistical Package for the Social Sciences (SPSS) version 20 was used for statistical analysis. Continuous variables are expressed as the means ± standard deviations when normally distributed and as medians (interquartile ranges [IQRs]) when not normally distributed. The Kolmogorov Smirnov Test was used to examine whether the data were normally distributed. If there was a normal distribution between the data, two group comparisons were made with the Student’s t-test, and if the data were not normally distributed, the Mann-Whitney U test was performed. Bonferroni correction was applied for multiple comparisons. Effect sizes were reported as Cohen’s d for Student’s t-tests and r for Mann-Whitney U tests. Multiple linear regression analysis was applied to determine the factors that were significant in the MRCI score. A full model with all factors included and a forward selection method model with only significant variables were established. Binary logistic regression models were created to determine factors affecting 30-day and 90-day rehospitalization. Significance and goodness of fit tests of the models were applied. Odds Ratio (OR) values and corresponding 95% confidence intervals were calculated in logistic regression models. The Chi-square test was used to compare categorical variables. p values less than 0.05 were considered statistically significant.

## Results

In this study 136 patients were included and of whom 63.2% were male. The mean (± SD) age was 75.29 ± 7.61 years. Most patients (75%) had polypharmacy and (91.2%) comorbidities.

The median number of medications used was 6 (IQR,4.25-9). The mean (± SD) MRCI score was 23.4 ± 13.65. 41% % of the patients were using anticholinergic drugs. Table 1 shows the sociodemographic and clinical characteristics of the patients.

**Table 1:** Socio-demographic and clinical characteristics of the patients

Variables	Total (*n* = 136)
Gender (%)	
Male	86 (63.2)
Female	50 (36.8)
Age (years) (mean ± SD)	75.29 ± 7.61
Previous hospitalization in the last 6 months (%)	34 (25)
Presence of comorbidity (%)	124 (91.2)
Hypertension (%)	83 (61)
Diabetes (%)	53 (39)
Thyroid (%)	12 (8.8)
Asthma (%)	9 (6.6)
COPD (%)	9 (6.6)
Neuropsychiatric diseases (%)	13 (9.6)
eGFR (mL/min/1.73 m2) (median-IQR)	13.5 (7.14–26.5)
Serum creatinine (mg/dL) (median-IQR)	3.47 (1.97–6.39)
Number of drugs (median-IQR)	6 (4.25-9)
Polypharmacy (Concurrent use of ≥ 5 drugs)	102 (75)
MRCI score	23.4 ± 13.65
Anticholinergic drug use (1 ≥ ACBS)	56 (41.2)
Rehospitalization within 30 days (%)	40 (29.4)
Rehospitalization within 90 days (%)	44 (32.4)

*SD* Standard deviation, *IQR* Interquartile range, *MRCI* Medication Regimen Complexity Index, *ACBS* Anticholinergic cognitive burden scale, *COPD* Chronic Obstructive Pulmonary Disease

Table 2 shows the relationships between patient clinical characteristics and MRCI scores. The MRCI score was significantly higher in patients with polypharmacy, anticholinergic drug use, diabetes and, chronic obstructive pulmonary disease than in those without polypharmacy (*p* < 0.05). Polypharmacy and anticholinergic drug use were found to be statistically significant with Bonferroni correction. The Cohen’s d value of polypharmacy was 1.462, indicating that polypharmacy had a very strong effect on MRCI score.

**Table 2:** The relationship between patient clinical characteristics and MRCI scores

Variables	MRCI scores	*p*-value	Effect size
Gender (median-IQR)			
Male	21 (14.75–31.25)	0.292	0.090
Female	17 (12.75–34.25)		
Age (years) (median-IQR)			
< 75	19 (13-34.5)	0.846	0.016
≥ 75	20 (15–30)		
Previous hospitalization in the last 6 months (median-IQR)	21 (16.75–37.25)	0.121	0.133
Rehospitalization within 30 days (mean ± SD)	26.90 ± 15.6	0.054	0.366
Rehospitalization within 90 days (mean ± SD)	27.02 ± 15.47	0.032	0.397
Polypharmacy (Concurrent use of ≥ 5 drugs) (mean ± SD)	27.63 ± 12.95	< 0.001*	1.462
Anticholinergic drug use (1 ≥ ACBS) (median-IQR)	26 (18.25-37)	< 0.001*	0.396
Presence of comorbidity (median-IQR)	20 (15-32.75)	0.057	0.163
Hypertension (median-IQR)	21 (15–31)	0.591	0.046
Diabetes (mean ± SD)	26.87 ± 14.96	0.018	0.423
Thyroid (mean ± SD)	30.08 ± 17.95	0.076	0.541
Asthma (mean ± SD)	26.89 ± 11.29	0.43	0.273
COPD	34.56 ± 22.14	0.011	0.893
Neuropsychiatric diseases	25 ± 11.54	0.659	0.129

Effect sizes are reported as Cohen’s d for t-tests and r for Mann–Whitney U tests

*SD* Standard deviation, *IQR* Interquartile range, *MRCI* Medication Regimen Complexity Index, *ACBS *Anticholinergic cognitive burden scale, *COPD* Chronic Obstructive Pulmonary Disease

*P values were significant according to Bonferroni correction

A multiple linear regression model was created to identify the factors affecting the MRCI scores of the patients. The overall significance level of the model was found to be high (*p* < 0.001). No multicollinearity or autocorrelation problems were detected in the model. In the full model in which all the variables were included, diabetes, thyroid diseases and hypertension had significant effects (*p* < 0.05). Diabetes, thyroid diseases and hypertension increased the MRCI score. As a result of the forward selection method applied to determine only significant factors, a significant effect of the variables ACBS score, diabetes, thyroid diseases, hypertension and rehospitalization within 90 days was found (*p* < 0.05) (Table 3).

**Table 3:** Full and forward regression models of factors affecting MRCI score

Factors (Full factorial)	Beta	Standard Error	t	*p*
Age (years)	-0.111	0.146	-0.760	0.449
Rehospitalization within 30 days	1.518	6.532	0.232	0.817
Rehospitalization within 90 days	6.635	6.283	1.056	0.293
Previous hospitalization in the last 6 months (%)	6.225	5.904	1.054	0.294
Diabetes mellitus	6.407	2.367	2.707	0.008
Thyroid	7.526	3.730	2.018	0.046
Hypertension	5.529	2.459	2.248	0.026
Asthma	1.002	4.695	0.213	0.831
COPD	6.365	4.388	1.451	0.149
Neuropsychiatric disease	2.494	3.660	0.681	0.497
Anticholinergic drug use (ACBS ≥ 1)	4.640	4.118	1.127	0.262
Presence of comorbidity (%)	1.852	4.170	0.444	0.658
ACBS score	4.143	2.188	1.894	0.061

Factors (Forward selection)	**Beta**	**Standard** **Error**	**t**	***p***
ACBS score	6.118	1.122	5.453	< 0.001
Diabetes mellitus	7.407	2.160	3.428	0.001
Thyroid	7.638	3.586	2.130	0.035
Rehospitalization within 90 days	5.321	2.207	2.411	0.017
Hypertension	4.884	2.267	2.155	0.033

*MRCI* Medication Regimen Complexity Index, *ACBS* Anticholinergic cognitive burden scale, *COPD* Chronic Obstructive Pulmonary Disease

Table 4 shows the logistic regression analysis of factors affecting rehospitalization. Only MRCI score and diabetes had a significant effect on rehospitalization within 30 days (*p* < 0.05). An increase in the MRCI score increased the number of rehospitalizations within 30 days by 1.04 (OR = 1.046, 95% CI = 1.007–1.081, *p* = 0.019), and diabetes increased the number of rehospitalizations within 30 days by 3.35 (OR = 3.35, 95% CI = 1.250–8.977 *p* = 0.016).

**Table 4:** Factors affecting rehospitalization according to logistic regression analysis

Factors affecting rehospitalization within 30 days				
Variables	Beta	*p*	OR	95% CI
Gender (Female)	0.168	0.716	1.183	0.478–2.932
Age (years)	-0.033	0.270	0.968	0.913–1.026
Previous hospitalization in the last 6 months (%)	1.245	0.239	3.472	0.438–27.542
MRCI score	0.042	0.019	1.043	1.007–1.081
Comorbidities	-2.060	0.076	0.127	0.013–1.238
Diabetes mellitus	1.209	0.016	3.350	1.250–8.977
Thyroid	1.473	0.094	4.363	0.779–24.425
Hypertension	-0.263	0.590	0.769	0.295–2.003
Asthma	0.398	0.633	1.488	0.291–7.605
COPD	-0.423	0.625	0.655	0.120–3.584
Neuropsychiatric disease	-0.339	0.614	0.713	0.191–2.658
Anticholinergic drug use (ACBS ≥ 1)	0.332	0.506	1.394	0.524–3.711

**Factors affecting rehospitalization within 90 days**				
**Variables**	**Beta**	***p***	**OR**	**95% CI**
Gender (Female)	0.200	0.654	1.222	0.509–2.932
Age (years)	-0.009	0.754	0.991	0.938–1.047
Previous hospitalization in the last 6 months (%)	1.192	0.243	3.295	0.445–24.406
MRCI score	0.045	0.010	1.046	1.011–1.083
Comorbidities	-1.941	0.093	0.144	0.015–1.380
Diabetes	1.117	0.022	3.056	1.178–7.929
Thyroid	1.069	0.174	2.911	0.623–13.597
Hypertension	-0.578	0.225	0.561	0.220–1.428
Asthma	0.401	0.624	1.494	0.301–7.420
COPD	-0.269	0.752	0.764	0.144–4.063
Neuropsychiatric disease	-0.142	0.831	0.868	0.235–3.200
Anticholinergic drug use (ACBS ≥ 1)	0.412	0.391	1.510	0.589–3.869

*MRCI* Medication Regimen Complexity Index, *ACBS* Anticholinergic cognitive burden scale, *COPD* Chronic Obstructive Pulmonary Disease

Similarly, significant effects of the MRCI score and diabetes were found in the factors affecting rehospitalization within 90 days (*p* < 0.05). An increase in the MRCI score was found to increase rehospitalization within 90 days by 1.04 times (OR = 1.046, 95% CI = 1.011–1.083, *p* = 0.01), and diabetes increased rehospitalization within 90 days by 3.05 times (OR = 3.056, 95% CI = 1.178–7.929, *p* = 0.022).

## Discussion

In this study, the ACBS score, diabetes, thyroid diseases, hypertension and rehospitalization within 90 days had a significant effect on the MRCI score. Diabetes and MRCI were factors affecting rehospitalization within 30 days and 90 days. Additionally, in this study, the mean (± SD) MRCI score was 23.4 ± 13.65.

A study carried out by Tesfaye et al. [9] in older adults with CKD patients, reported that the median MRCI score was 27 (IQR, 20–35), and in a study conducted by Parker et al. [33] in older adults with CKD, the median MRCI score was reported to be 22.8 ± 7.7. There is no definitive cut-off point for MRCI scores [20]. While some studies evaluate it as a continuous variable, as in this study [20, 34] other studies have variable cut-off values between 8 and 35 [18, 35–37]. Abdelbary et al. examined the association of medication complexity with rehospitalization in heart failure patients and reported that an MRCI score greater than 15 was considered highly complex [21]. In a validation and standardization study of the MRCI score for older adults in primary care in Brazil, MRCI scores below 9 were classified as low, 9–16.5 as moderate, and 16.5 or higher as high [38]. Although the MRCI score in this study was high, it is within the score range reported in the literature. The MRCI scores in this study were quite similar to those reported in the study conducted by Parker et al., but slightly lower than those reported in the study conducted by Tesfaye et al. [9]. This may be because the mean patient age in this study, while similar to that of Parker et al. [33], was younger than the patient population in Tesfaye et al.‘s study [9].

According to research carried out by Parker et al. in older adults with CKD patients, female gender, Charlson comorbidity index of 4 or 5, and various phosphate binders were found to be associated with the MRCI score [33]. In this study, as in most studies conducted on this subject, no relationship was found between gender and the MRCI score [17, 38, 39]. In a population-based study on elderly patients, having a higher prevalence of pain, having more comorbidities, and not living at home were factors influencing a higher MRCI [40]. In a study performed in primary care, high MRCI was associated with diabetes and asthma/chronic obstructive pulmonary disease [38]. Research undertaken in hospitalized patients showed a relationship between the number of medications used and MRCI scores [41]. A study conducted at the community pharmacy, the presence of polypharmacy, the presence of comorbidities, and having less than eight years of education were associated with significantly higher MRCI scores [39]. Although differences were observed in the mentioned studies due to different patient populations, treatment regimens and study designs, it was understood that the MRCI score was associated with comorbidities and polypharmacy.

In this study, the ACBS score, diabetes, hypertension and thyroid disease were found to be associated with an increase in the MRCI score. Diabetes was a comorbidity that contributes particularly to MRCI. Studies have reported a high prevalence of MRCI scores in diabetic patients, and poor glycemic control has been associated with a high MRCI score [42, 43].

This may be because diabetes requires multiple medications, different dosage forms, and instructions. In this study, the addition of insulin to the treatment regimen may have increased medication complexity due to its injection administration [44]. In this study, hypertension was found to be associated with a higher MRCI score, similar to the findings of previous studies.

Additionally, patients with diabetes often also had hypertension, which may have influenced the increase in MRCI scores [45, 46]. To our knowledge no study has specifically investigated the relationship between thyroid disease and MRCI, but there is one study that has shown that thyroid disease also increases the MRCI score [39]. In this patient population, the number of patients with thyroid diseases was small (*n* = 12) and the MRCI score may have been high due to the thyroid patients having other comorbidities.

Identifying the relationship between MRCI and rehospitalization is important. Rehospitalization imposes a financial burden on the healthcare system and an emotional burden on patients and caregivers [13]. As reported in a study by Tesfaye et al. in older adults with CKD patients, unlike this study, no relationship was found between MRCI scores and rehospitalization within 30 days [9]. Previous studies involving patients with CKD revealed that, patient factors, comorbidities, the number of previous hospitalizations, and the length of hospital stay are among the factors affecting rehospitalization [9, 13]. In this study, diabetes and the MRCI scores were found to be associated with rehospitalization within 30 and 90 days. In their study, Tesfaye et al. did not examine comorbidities (e.g., diabetes, asthma, etc.) individually; they used only the Charlson comorbidity index. The reason why the MRCI score was not associated with rehospitalization in the study conducted by Tesfaye et al. may be that the patient population was very old and that rehospitalization was affected by other factors and suppressed the MRCI score. The very old adult patient population is already at high risk of rehospitalization, regardless of medication complexity [47]. Rehospitalization rates are high in patients with diabetes [48]. Diabetic patients experience numerous acute and chronic complications. Increased risk of infection, advanced age, multiple medication use, and insulin use contribute to rehospitalizations in diabetic patients [49, 50]. Diabetes, thyroid diseases and hypertension increased the MRCI score, but many other risk factors affecting rehospitalization, such as patients’ socioeconomic, psychosocial, and treatment compliance, were not evaluated in this study [48–51].

In this study, although the ACBS score was one of the factors affecting the MRCI score, it did not affect rehospitalization. Few articles have considered anticholinergic burden and medication complexity together Tesfaye et al. used the modified anticholinergic risk scale (mARS) to determine anticholinergic drug use in their study of older adult patients with CKD.

They reported no association between anticholinergic medication use and rehospitalization [17]. As the anticholinergic drug burden increases, complications such as delirium, and falls increase, and consequently, rehospitalization may increase [52]. However, the relationship between the anticholinergic drug load and rehospitalization rate is unclear. Some studies reported a significant relationship [26, 52, 53], whereas others did not [54, 55]. Differences in the scales used, study design, sample sizes and follow-up periods may be the reasons for the differences in results between studies.

In studies investigating the relationship between MRCI and rehospitalization, the results were variable [18, 21, 34, 56]. In a systematic review and meta-analysis, MRCI scores were associated with rehospitalization and medication adherence [18]. In studies investigating the relationship between MRCI scores and rehospitalization in heart failure patients, a study by Colavecchia et al. [56] reported a relationship between MRCI and rehospitalization, whereas no relationship was found in a study by Abdelbary et al. [21]. Colavecchia et al.‘s [56] study in the United States included a larger cohort (1452 patients) of all patients over the age of 18, encompassing both black and white patients. Abdelbary et al.‘s [21] study in Qatar included a more modest patient population (150 patients) over the age of 65. The difference in patient sample size, the heterogeneity of the patient group, and differing healthcare systems may have contributed to this difference.

A prospective study conducted in a geriatric assessment and management unit reported no associations between MRCI scores and rehospitalization [20]. Additionally, in a multicenter study of patients hospitalized for heart failure, acute myocardial infarction, pneumonia, and chronic obstructive pulmonary disease, the rehospitalized group had higher MRCI scores than the non-rehospitalized group. However, models controlling for the MRCI showed no significant effect [34]. From this study, it can be concluded that the clinical effect of medication complexity may be weaker in patients with high comorbidities. Differences between studies may be due to study design, patient population and socioeconomic factors, and physician prescribing habits [10, 57, 58].

There are limited studies on medication complexity in Turkey. In two studies conducted with older adult patients in a pharmacy setting, one reported a median MRCI score (IQR) of 12.5 (7–19.6), and the other reported a score of 11 (7–15) [39, 59]. These studies did not examine the relationships between MRCI scores and clinical outcomes. The lower MRCI scores in the pharmacy setting may be due to the frailty and higher comorbidity levels of older adult patients in the hospital setting. One study was conducted in the medical oncology clinics of a tertiary hospital among cancer patients over the age of 18. The median MRCI scores at discharge were higher in patients with unplanned hospitalizations within 30 days than in those without hospitalizations [60]. Our study is of clinical importance given the limited number of studies on this topic in Turkey.

On the basis of this study, medication regimens in older patients with CKD can be simplified by using simpler dosage forms and longer-acting, lower-dose medications. When clinically appropriate for the patient, drug forms such as tablets or capsules should be preferred. The patient’s medication regimen should be reviewed, and unnecessary medication use should be avoided. Additionally, patient adherence to the medication regimen should be assessed. These factors are particularly important for older patients with cognitive impairment who may struggle with adherence. Healthcare professionals should consider these factors when prescribing medications to older adults with CKD.

### Strengths and limitations

This study had several limitations. These limitations were that it was conducted in a single center with a limited number of patients and that it was retrospective. Therefore, the results of this study cannot be generalized to all older adults with CKD. Owing to its retrospective nature, patient medication adherence and other geriatric syndromes could not be evaluated.

Data were collected from a single hospital. Therefore, patients’ hospitalizations to other hospitals within 30 and 90 days were not recorded. Although many variables identified as being associated with MRCI and 30- and 90-day rehospitalizations were collected in this study, some sociodemographic variables, patients’ OTC medications, and clinical information were not available due to the retrospective nature of the study. Patients’ socioeconomic level marital status, family support, and literacy may influence patient adherence and, consequently, rehospitalization. Despite this, the use of a validated tool to calculate medication complexity and the assessment of anticholinergic burden were the strengths of our study.

### Recommendations for future research

Future studies need to be conducted in a multicenter, large population and prospectively.

Additionally, patients’ medication adherence should be assessed, and sociodemographic data should be collected in greater detail. Even with high medication complexity, patients with higher patient adherence may exhibit better clinical outcomes, and whether this will reduce rehospitalization warrants further investigation Further studies on reducing medication complexity, including before-after and randomized controlled trials, are important for evaluating clinical outcomes.

## Conclusion

In this study, MRCI scores were found to be high. ACBS score, diabetes, thyroid diseases, hypertension and rehospitalization within 90 days had significant effects on the MRCI score.

Diabetes and MRCI scores were factors affecting rehospitalization within 30 days and 90 days. Reducing medication complexity can be a factor that may prevent rehospitalization in older adults with CKD.

## Data Availability

The data that support the findings of this study are available from the corresponding author upon reasonable request.
